# Nondestructive Determination of Strength of Concrete Incorporating Industrial Wastes as Partial Replacement for Fine Aggregate

**DOI:** 10.3390/s21248256

**Published:** 2021-12-10

**Authors:** Temple Chimuanya Odimegwu, A. B. M. Amrul Kaish, Ideris Zakaria, Manal Mohsen Abood, Maslina Jamil, Kayode-Ojo Ngozi

**Affiliations:** 1 Department of Civil Engineering, Infrastructure University Kuala Lumpur, Kajang 43000, Malaysia; odimtemplec@yahoo.com (T.C.O.); ideris@iukl.edu.my (I.Z.); dr.manal@iukl.edu.my (M.M.A.); 2 Department of Civil Engineering, Universiti Kebangsaan Malaysia, Bangi 43600, Malaysia; 3 Department of Architecture & Built Environment, Universiti Kebangsaan Malaysia, Bangi 43600, Malaysia; maslinajamil@ukm.edu.my; 4 Department of Civil Engineering, University of Benin, Benin City 1154, Nigeria; Ngozi.kayode-ojo@uniben.edu

**Keywords:** compressive strength, nondestructive test, Schmidt rebound hammer test, fine aggregate, industrial waste

## Abstract

Schmidt rebound hammer test was employed in this study as a nondestructive test. This test method has been universally utilized due to its non-destructiveness for quick and easy assessment of material strength properties and quality of concrete of an existing structure. Industrial waste materials (air-dried alum sludge, treated alum sludge, limestone dust and quarry dust) were employed as replacement material for fine aggregates in this study. A normal strength concrete was designed to achieve 35 MPa at 28 days, with industrial waste materials replacing fine aggregate at different percentages (0%, 5%, 10% and 15%), and then cured for 7, 28 and 180 days. The compressive strength values and rebound numbers for all the mixes obtained were correlated, and a regression equation was established between compressive strength and Schmidt rebound number. The correlation result showed an excellent relationship between rebound number and compressive strength of concrete produced in this study at all curing ages, with correlation coefficients of R^2^ = 0.98, R^2^ = 0.99 and R^2^ = 0.98. The predicted equation showed a strong relationship with the experimental compressive strength. Therefore, it can be used for the prediction of compressive strength of concrete with industrial waste as a replacement for fine aggregate.

## 1. Introduction

Nondestructive testing has been widely used in the construction industry to test the properties (strength properties) of materials or structures, the differences in structural defects and characteristics, and the quality of materials and structures during and after construction and within the service life of the structure. This system of testing has been proven to be easy, quick and still gives a reliable result of the tested concrete structure or sample. Above all, it serves various testing purposes beyond being an effective testing tool for inspection and measuring of the strength and quality of hardened concrete or an existing concrete structure [1]. Rebound hammer has been used in the past to measure the compressive strength of concrete and establish the quality of concrete structures. It is also employed to establish the characteristics of material properties and evaluation of structural defects for existing structures due to time and environmental factors. These tests are usually done without causing internal or physical damage to the structure or product; ideally, the test method is cheaper and less time-consuming [2]. However, the nondestructive test becomes very important for structures that are already existing or a building that needs maintenance which cannot be achieved by the destructive test methods [3] due to several aspects such as preservation of samples or structure, simplicity and flexibility. Hence, this test method is rated as dependable [4,5] for evaluating the strength properties of existing engineering materials and structures (metals, rocks, concrete pavements, bridges and buildings). Since there is no sample loaded directly until failure occurs, the strength of materials using the nondestructive test is derived based on estimation, and no absolute strength value is provided [6]. Schmidt hammer hardness test (rebound hammer number) and ultrasonic pulse velocity (UPV) are the common nondestructive tests used in several construction industries. Numerous studies have underscored the reliability of the nondestructive test in terms of assessing the mechanical properties of materials and existing building structures [7,8,9]. Correlation and prediction of compressive strength of materials or structures have been studied previously [3,4,10,11]. The results from these studies showed a good relationship between compressive strength and the nondestructive test values (UPV values and rebound hammer number) and can be useful in testing in situ compressive strength and prediction of the strength of structures. Different precise studies have been done using Schmidt rebound hammer test, such as evaluation of rock weathering [4,12,13], uniaxial compressive strength of rock [14], prediction of durability and compressive strength of limestone rocks [15], and relationship between unconfined compressive strength and Schmidt rebound number of gypsums using empirical equation [16]. Evaluation of concrete structures has also been reported using the Schmidt rebound hammer test and UPV test [5]. The findings affirmed that the rebound hammer test is found to depend on the properties of the concrete mix. Even when compared to another nondestructive test such as UPV, the Schmidt hammer test is seen to have a better correlation than UPV [17]. Another study confirmed that the rebound hammer test was discovered to be somewhat reliable after a study was done on strength and safety assurance on an existing structure [18]. Studies have proven that nondestructive testing is important in most cases where drilling of the concrete core is not advisable, because of the weakening of concrete structure due to drilling [19]. The drilling process when obtaining concrete core samples from an existing structural member will cause a reduction in the load-bearing capacity of the concrete, thus affecting the integrity of the concrete structure and possibly causing a structural defect in the future [20].

Nevertheless, it is important to understand that several factors should be in place when evaluating concrete structures using the Schmidt rebound hammer test. Factors such as the surface irregularity of sample, space between impact, surface hardness, age of the concrete sample, moisture of sample, presence of void and calibration of Schmidt rebound hammer [3,13,20,21] are used to evaluate the relationship between nondestructive (Schmidt rebound hammer test) and destructive test on compressive strength of concrete. It was reported that the correlation coefficient showed an excellent relationship between rebound number and compressive strength. High compressive strength yields a high rebound number and low compressive strength yields a low rebound number. This proves that rebound number is relevant in the prediction of compressive strength of concrete [21]. Accuracy and reliability of rock blocks of different strengths have been studied [11], with rebound number used to evaluate the empirical relationship, using multiple regression analysis. It was asserted that this method is proposed to determine the uniaxial compressive strength of the existing rock. This finding is similar to that of a study by Rahmouni et al. [22]. Another combined method of nondestructive testing was employed to investigate the compressive strength of existing concrete, using Schmidt hammer and UPV for comparison with a concrete cube at different ages (28 and 90 days). It was found that the Schmidt hammer gave a more reliable result in the prediction of compressive strength than the result from the UPV test method [23]. Several studies have indicated that the Schmidt hammer test method produces a more accurate result when used as means to predict the compressive strength of the concrete and existing concrete structure. Moreover, the Schmidt hammer test used for the prediction and estimation of compressive strength of concrete is suitable [24], as the compressive strength value of the Schmidt hammer is almost similar to the actual compressive strength values from concrete crushing strength. Liu et al. [25] stated that when using a nondestructive test to determine or predict compressive strength, a correlation coefficient of 0.9622 could be achieved. In effect, this is an indication that the proposed method (nondestructive method) has a strong relationship with the compressive strength. Feasibly, this approach can be used in developing a nondestructive test to predict compressive strength. Schmidt rebound number versus compressive strength and ultrasonic pulse velocity versus compressive strength were studied, and the correlation between two different models of nondestructive test method was compared [26]. It was affirmed that the correlation between Schmidt rebound number versus compressive strength gave more accurate result values than the model of ultrasonic pulse velocity versus compressive strength.

Evaluation of compressive strength and other strength properties of concrete using conventional direct tests such as compressive strength testing (using a compressive machine) is expensive, labor-intensive and time-consuming. On the other hand, reported studies have shown that the Schmidt rebound hammer test is reliable in the prediction of concrete strength when used in the correlation between Schmidt rebound hammer result and compressive strength of concrete. However, there are limited studies on concrete samples produced with some industrial waste materials (alum sludge, quarry dust, and limestone dust) tested with the nondestructive test method. In this study, the Schmidt rebound hammer was used as a nondestructive test to measure and predict the strength of concrete samples with and without industrial waste material as a replacement for fine aggregate. However, some past studies employed different industrial waste as replacement material to improve the performance of concrete structures [27,28,29]. Durability properties of concrete utilizing alum sludge as a replacement for cement were studied by Breesem et al. [30]. It was reported that alum sludge as a replacement for cement had no negative effect on the concrete structure but had higher resistance to chemical attack. Alum sludge in different conditions utilized in concrete as a replacement for fine aggregate has been studied [31]. The study disclosed that alum sludge as a replacement for fine aggregate increased the concrete density and strength properties and improved concrete durability. Quarry dust has also been utilized as replacement material in concrete. It was reported that quarry dust as a replacement for fine aggregate in concrete increased the strength of concrete tremendously by up to 8–20% [32]. Hamid et al. [29] highlighted that the addition of quarry dust above 50% as replacement material will yield a negative effect on concrete compressive strength and workability. Experimental investigation of industrial waste utilized in concrete production showed significant improvement in the concrete properties [33,34,35], representing a sustainable approach towards the compliance with prospect needs of environmental and concrete technology [36]. Omar et al. [37] utilized limestone waste as a replacement material in concrete. It was reported that addition of limestone improves the concrete workability and compressive strength. Furthermore, it is important to comprehend that the excessive exploitation and consumption of natural resources such as river sand due to the increase in demand for natural river sand have caused some of these natural resources to be depleted and increase in cost with time. Moreover, with time, these activities result in increased depth of river bed, erosion, disturbance of aquatic life and lowering of the water table. It is, thus, imperative to consider the importance of utilizing industrial waste materials (alum sludge, quarry dust and limestone dust) as a replacement for fine aggregate for sustainable development of greener concrete production.

The use of industrial waste materials such as alum sludge, quarry dust and limestone dust will reduce the excessive consumption of natural river sand, which is being depleted with time. Most studies from the literature focused on existing reinforced structures [17,19,23] and materials such as weathered rock [4,13], granite and bricks [3,26], while in this study, the experimental studies on laboratory-produced concrete materials made with industrial waste materials such as alum sludge, quarry dust and limestone dust are the focus. The aim is to determine the compressive strength of the produced concrete using a nondestructive approach. Moreover, it is clear from the reviewed literature that little or no study has been done on compressive strength determination of concrete with alum sludge, quarry dust and limestone dust as replacement for fine aggregate using the Schmidt rebound hammer test method (nondestructive test method). It is necessary to study the effect of industrial waste material as fine aggregate on the rebound hammer test and the relationship between compressive strength and rebound number. This study contributes towards bridging this gap in research.

### Novelty/Contribution to Knowledge

From the available literature, there is limited experimental study on the use of alum sludge and other industrial waste material as a replacement for fine aggregate to determine concrete strength using a nondestructive method of testing. Thus, this research provides an alternative choice for concrete testing using a nondestructive method (Schmidt rebound hammer test) to predict the compressive strength of concrete, with formulas derived from this study. This study contributes to the prediction of compressive strength of existing concrete with the empirical formula generated using the Schmidt rebound hammer test method. The use of Schmidt rebound hammer tests has been limited in the evaluation of concrete with alum sludge and other industrial waste materials as a replacement for fine aggregate. Thus, it is paramount to have a well-detailed experimental investigation on this industrial waste material using a nondestructive test method. In addition, this study provides further knowledge on the behavior of industrial waste materials introduced in concrete mix and the suitable proportion of these materials to produce concrete with better strength when compared to the control concrete sample. Invariably, the result from this study proves that the Schmidt rebound number can be used for the prediction of compressive strength even when new materials are introduced to the concrete mix. 

## 2. Materials and Methods

### 2.1. Materials

The cement used in this study is ordinary Portland cement that satisfied the specification for ordinary Portland cement according to ASTM C-150 [38]. The fine aggregate was graded river sand with particles passing 4.75 mm BS sieve conforming to BS 882:1992 [39]. Crushed stone aggregate from the quarry industry (Kajan quarry) with various particle sizes complying with BS 882:1992 [39] and ASTM C33/C33M-18 [40] was utilized as coarse aggregate. The water used was treated tap water that is fit for drinking and does not contain particles that might affect the process of hydration of the cement or the behavior of the concrete and conforms to the specification of BS 3148:1980 [41]. Alum sludge from the drinking water treatment plant is a waste generated during the purification process of drinking water. The water treatment plant waste was collected from one of the treatment plants in Selangor, Malaysia, located at Putrajaya (Sungai Semenyih water treatment plant). Thereafter, it was kept in the metallic tray for complete air drying. Another part of the sludge was treated in a furnace under the temperature of about 200 °C for 3 h to remove any organic materials that could affect the concrete. The alum sludge was then crushed with a Los Angeles Abrasion Testing Machine and sieved to a desired size close to sand. A similar size was also achieved by sieving for both limestone dust and quarry dust materials used in this study.

### 2.2. Physical Properties of Materials

The physical properties of all materials utilized for the production of concrete are presented in Table 1. The coarse aggregate used in this study has a specific gravity of 2.75 and an absorption rate of 0.54%. The fine aggregate is river sand with a specific gravity of 2.62 and a water absorption of 0.76%. Limestone dust used as a replacement for fine aggregate had a specific gravity of 2.57 and an absorption rate of 1%; it was similar to quarry dust, which had a specific gravity of 2.58 and an absorption rate of 1.10%. The result shows that both alum sludges had the lowest values for all the test values, with specific gravity values of 2.38 and 2.35 and water absorption rates of 12.52% and 12.5%; a similar result was found in a study by Kaish et al. [42]. 

### 2.3. Chemical Properties of Materials

The chemical composition of all the materials as fine aggregate is presented in Table 2. It is seen that the fine aggregate (river sand) is rich in silica as its main element with a concentration of 80.96%. Alum sludge in both air-dried and treated conditions showed little difference in chemical composition. It is seen that both alum sludge conditions have silica and aluminum oxide as their main chemical elements. Air-dried alum sludge contains 41.98% silica and 33.09% aluminum oxide, while the treated alum sludge contains 42.74% and 33.28% aluminum oxide. This indicates that the treatment of alum sludge at low temperature had little effect on the chemical concentration. There were other elements such as CaO, Fe_2_O_3_, K_2_O and MgO in both alum sludges in low concentrations. For limestone dust, the main element present is calcium oxide, with a concentration of 77.8%, and other elements are present in low concentrations, while quarry dust had silica as its main element with a concentration of 66.8%. It is seen that there are other elements present with very low concentration as shown in Table 2.

### 2.4. Mix Design

**Figure 1:**
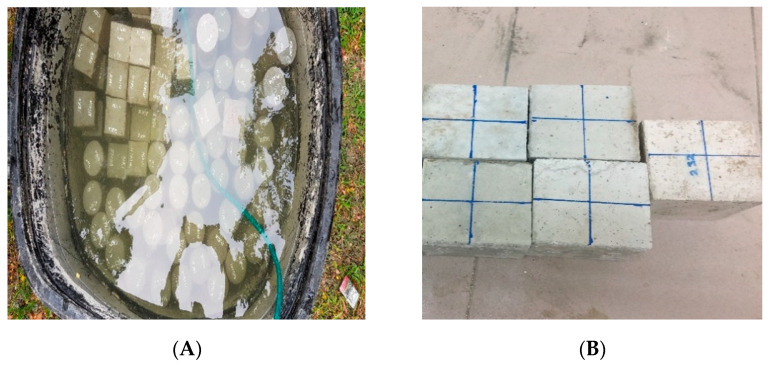
Concrete samples in curing tank and samples marked for rebound hammer test. (**A**) Concrete samples in curing tank. (**B**) Concrete samples prepared for Rebound hammer test.

A normal concrete design of 35 MPa according to British Standard BS 8500, 2006 [43], was adopted. The conventional constituent materials (ordinary Portland cement, fine aggregate and crushed coarse aggregate) were used for control concrete mix. A design concrete slump of 30 to 60 mm and a constant water–cement ratio of 0.52 were adopted, as shown in Table 3. Constant water content was employed in all batches of the concrete mix to identify the change in workability. Alum sludge in both conditions (air-dried and treated), quarry dust and limestone dust were used as replacement materials for fine aggregate in replacement percentages of 0%, 5%, 10% and 15%, respectively. Different batches were employed in the mix, and the reference sample was concrete mix without industrial waste materials (as in Table 3). However, due to the absorption nature of alum sludge, additional water was calculated for air-dried and treated alum sludge. The additional water was calculated based on the water absorption capacity of both alum sludges as presented in Table 1. The concrete was cured conventionally in water at ages of 7, 28 and 180 days (see Figure 1A). The considered concrete size for the destructive compressive strength test and Schmidt rebound hammer test was a 100 mm cube (see Figure 1B).

### 2.5. Rebound Hammer Test on Concrete

**Figure 2:**
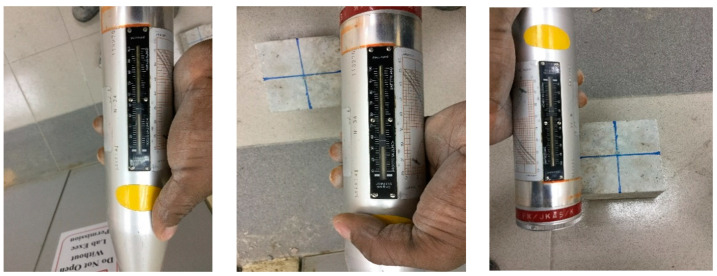
Rebound hammer reading after testing on concrete sample using rebound hammer method.

First, several factors were considered before performing the test. A calibration test was performed on the rebound hammer by testing it against the test anvil to have a dependable result, as the manufacturer of this test equipment has indicated. After the calibration was done, the surfaces of the testing concretes were smoothened to have a smoother surface to test on and avoid surface irregularity that could affect result accuracy. The samples were also allowed to dry so that the concrete sample would be free of moisture, which might affect the reading of the Schmidt rebound number. Then a light pressure was applied on the plunger, which released it from its lock position and thereby allowed it to extend to the test position which is a zero reading. After that, the plunger was pressed against the surface of the testing concrete specimen, keeping the rebound hammer perpendicular to the surface of the concrete. Then, a gradual increase in pressure was applied until the hammer impacted a hammer blow, and the plunger was locked to take the reading by pressing the bottom at the side of the equipment. This test was performed on three concrete cubes about 16 times (four times for each cube). One sample had about four blows of the rebound hammer until a consistent reading was achieved; thereafter, an average of the reading was taken (see Figure 2). Table 4 presents the interpretation of the rebound hammer test results.

The test was performed as specified in BS 1881, part 202 [44], for the nondestructive method of testing for concrete Part 4, by surface hardness method. Finally, after the Schmidt rebound hammer test was done, three samples for each mix (total of 117 samples) at different curing ages (7, 28 and 180 days) were moved to the universal testing machine for the compressive strength test. The result of the compressive strength was used to study the correlation between compressive strength and rebound number.

## 3. Results and Discussion

### 3.1. Compressive Strength of Concrete

**Figure 3:**
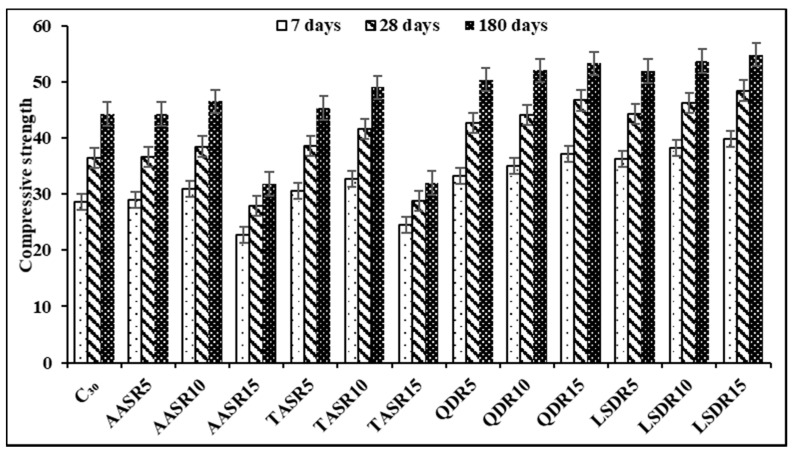
Compressive strength of concrete with industrial waste material as fine aggregate.

The destructive method of compressive strength testing was used to determine the strength of concrete produced without industrial waste (control) and with industrial waste (air-dried and treated alum sludge, limestone dust and quarry dust). The graph presented in Figure 3 shows that the compressive strength of all the samples increased with an increase in curing age (7, 28 and 180 days) [31]. The strength of the concrete increased when industrial waste material was added to the mix, and when the waste was increased the compressive strength increased too. Except for concrete with alum sludge at replacement increase up to 15% (AASR15 and TASR15), it was observed that the strength decreased when compared to the control sample C_30_, while quarry dust in all replacement contents yields an increased strength with an increase in replacement content.

The result for concrete produced with quarry dust showed an increase in compressive strength at all curing ages as replacement content increased; this behavior was different when compared with alum sludge. It was also observed that quarry dust had a better strength result than alum sludge. Figure 3 shows an increase in compressive strength with an increase in limestone dust content in all fine aggregate replacement percentages. Limestone dust in all replacement percentages had better compressive strength performance than all other industrial waste materials employed in the study as replacements for fine aggregate. LSDR15 had the highest compressive strength at all curing ages when compared to other materials used as replacement of fine aggregate. This increase in strength is similar to the findings reported by Shuhua and Peiyu [45]. Furthermore, the rate of increase in compressive strength for all percentages of limestone dust as fine aggregate replacement was high between 7 and 28 days. Meanwhile, when the curing age progressed, the rate of increase was lower, and this behavior was also found with the quarry dust content in all replacement percentages. This agrees with the findings by Kaish et al. [10], which indicate that the strength increase is mainly due to the effect of the particle fineness of the replacement materials which reduced the internal void in the concrete and yielded to an increase in concrete strength.

### 3.2. Effect of Industrial Waste Materials on Rebound Hammer Numbers

**Figure 4:**
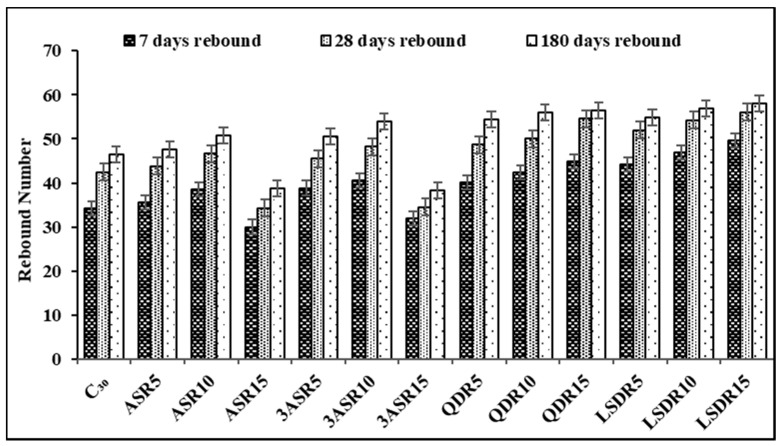
Average rebound number for different replacement contents and curing ages.

The result of the Schmidt rebound hammer test is presented in Figure 4. The result from the graph shows that the rebound number value increases with curing age in all percentages of replacement of fine aggregate with industrial waste materials (air-dried alum sludge, treated alum sludge, quarry dust and limestone dust) employed in this study. The behavior from the result also indicates that the rebound number increased with an increase in industrial waste content for all fine aggregate replacement contents except for increased content of AASR15 and TASR15. This indicates that the increase in alum sludge content up to 15% as a replacement for fine aggregate decreases the rebound number. When the behaviour of concrete sample AASR15 and TASR15 subjected to rebound hammer test was compared with the behaviour of same sample AASR15 and TASR15 subjected to compressive strength, it was seen that AASR15 and TASR15 had the lowest compressive strength and rebound number value. Similar evidence was highlighted by Samson and Moses [21], and it is seen that higher compressive strength produces higher rebound number value and lower compressive strength produces lower rebound number value.

The result of quarry dust as a replacement for fine aggregate presented in Figure 4 shows that the Schmidt rebound number increases with an increase in quarry dust at all curing ages. When compared to C_30_ and both concrete with alum sludge contents, it is seen that result for concrete with quarry dust was higher. This could also be attributed to their compressive strength performance (destructive test method). For limestone dust as a replacement for fine aggregate, it was found that the rebound number increased with an increase in limestone content. This behavior was found at all curing ages with limestone dust content. Among all industrial waste materials used in this study as replacements for fine aggregate, limestone dust had the best rebound hammer value. This behavior was similar to the performance of the concrete in compressive strength (destructive test method) based on the characterization of concrete quality as specified in BS:1881, part 202 [44], for rebound hammer measurement (Table 1). The quality of the produced concrete with air-dried and treated alum sludge can be characterized ranging from fair layer to very good hard layer with rebound numbers of 30 to 54, respectively, for AASR15 to TASR10 at 7 and 180 days of curing. C_30_ with rebound numbers above 30 and 40 is designated to be from a good layer to a very good hard layer. Quarry dust and limestone replacement for fine aggregate had a rebound number above 40 and was graded as very good hard layer concrete. 

#### Relationship between Compressive Strength and Rebound Number

**Figure 5:**
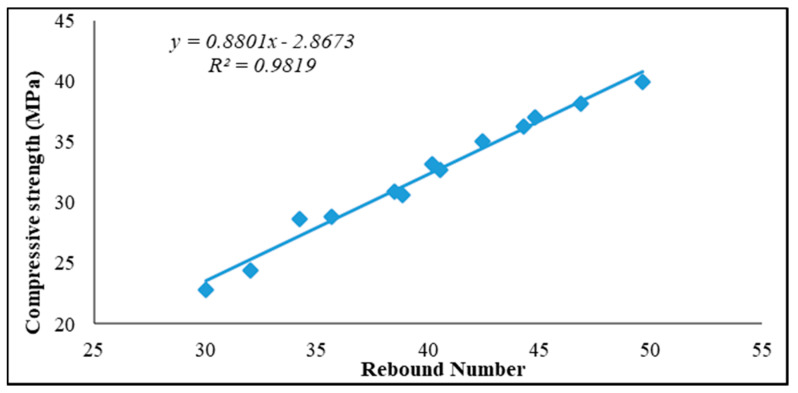
Relationship between rebound number and compressive strength of concrete aged 7 days.

**Figure 6:**
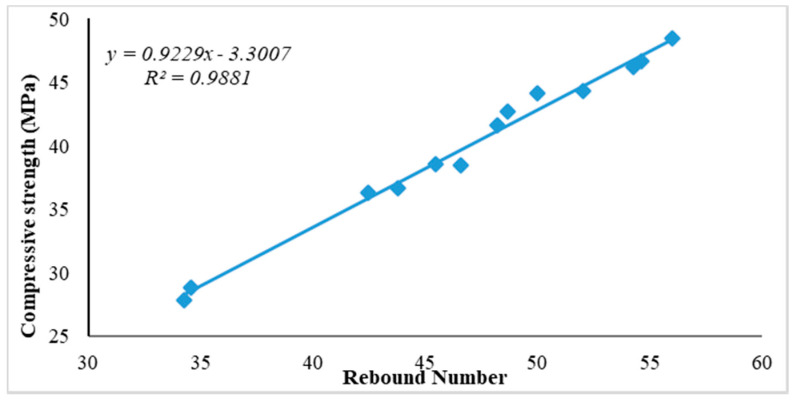
Relationship between rebound number and compressive strength of concrete aged 28 days.

**Figure 7:**
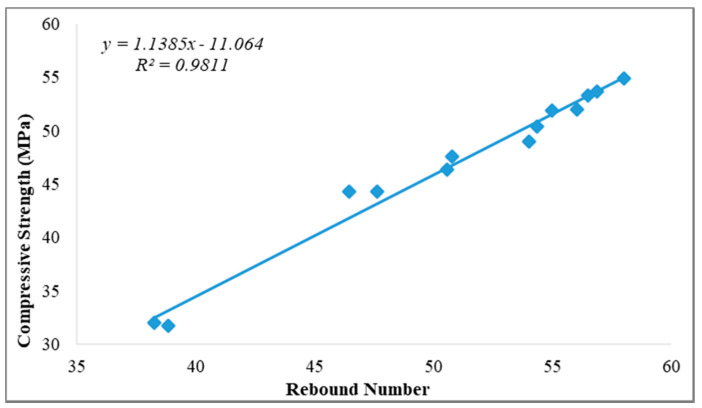
Relationship between rebound number and compressive strength of concrete aged 180 days.

The relationship between compressive strength and rebound number for all ages is presented graphically in Figure 5, Figure 6 and Figure 7 for curing ages of 7, 28 and 180 days, respectively. Two concrete cube samples of 100 mm were used to measure the average rebound number for each concrete sample and also used to establish its relationship with compressive strength for all ages and replacement contents. Linear correlation was used for this purpose in previous studies [3,26]. Rebound number has been used to predict compressive strength using an empirical equation to correlate between rebound number (*R*_n_) and compressive strength (*f_c_*); these adopted empirical formulas from past studies are presented in Equations (1)–(4) [26]. The empirical formulas recommended from past studies for the prediction of compressive strength using rebound number are expressed in Equations (1)–(4).

*f_c_* = 0.8*R*_n_ − 5.017 (1)

*f_c_* = 1.933*R*_n_ − 51.62 (2)

*f_c_* = 0.784*R*_n_ − 5.06 (3)

*f_c_* = 0.825*R*_n_ − 2.33 (4)

Equations (1)–(4), adopted from Aliabdo and Elmoaty [26], appeared to have different empirical formulas due to the fact that the investigation was on the individual behavior of different rocks (marble, white limestone, basalt, pink limestone) and building materials (bricks) from different sources. All the materials were tested independently and different formulas were generated from different materials and compared. The predicted formulas generated from Aliabdo and Elmoaty [26] were utilized in this study to compare the predicted compressive strength generated from this study with the predicted compressive strength generated using the proposed equation [26]. 

Figure 5, Figure 6 and Figure 7 present the experimental investigation on the correlation between rebound number and compressive strength of all the produced concrete samples used as replacement for fine aggregate that was cured at 7, 28 and 180 days.

The presented relationship graphs in Figure 5, Figure 6 and Figure 7 show a strong relationship between rebound number and compressive strength of the produced concrete for all curing ages, with 7, 28 and 180 days having correlation coefficients of R^2^ = 0.98, R^2^ = 0.99 and R^2^ = 0.98 respectively. Thus, the equation from the relationship graph result in Figure 6 (28 days curing age for all the concrete samples) was used for the prediction of concrete compressive strength. The empirical formula adopted from the relationship graph (Figure 6) for the prediction of compressive strength from the experimental rebound number was derived using the empirical equation given in Equation (5).

*f_c_* = 0.923*R*_n_ − 3.301 (5)

where, *R*_n_ = rebound number and *f_c_* = compressive strength of concrete (MPa)

Table 5 shows the correlation between rebound number and compressive strength obtained from the performed statistical analysis for the determination of theoretical values of compressive strength using the formulas derived from a past study from Equations (1)–(4) and formula from this study (Equation (5)).

The suggested equation from this study (Equation (5)) showed a very close relationship with the derived theoretical compressive strength (*R_nt_*) when compared with experimental compressive strength (*f_c_*), having a similar strength value for all fine aggregate replacement contents. The derived theoretical compressive strength from Equation (4) (empirical formula suggested by Aliabdo and Elmoaty [26]) was also found to have a compressive strength value similar to the experimental compressive strength (*f_c_*) and Equation (5). This shows that the suggested empirical formula from this study (Equation (5)) can be used for the prediction and estimation of compressive strength from rebound number. 

Another equation suggested by Aliabdo and Elmoaty [26] from Equations (1)–(3) showed a lower compressive strength when compared with the experimental compressive strength (*f_c_*). Thus, the suggested empirical formula from Equation (2) showed compressive strength that is somewhat similar to experimental compressive strength (*f_c_*). The result from Equation (2) showed a low prediction value at a higher rebound number and a low compressive strength at a low rebound number. The results from Table 5 also proved that when the experimental compressive strength increases, the theoretical compressive strength increases. Nevertheless, the ratio of experimental compressive strength and theoretical compressive strength (*R_nt_/f_c_*) from Table 5 showed that when rebound number *R*_n_ increases, the ratio of theoretical compressive strength (*R_nt_/f_c_*) increases with an increase in concrete compressive strength (*f_c_*) for the predicted empirical formula used and all percentages of replacement of fine aggregate with different industrial waste materials (5%, 10%, 15%). Similar behavior in correlation was observed in studies by Roknuzzaman et al. [3] and Aliabdo & Elmoaty [26]. The predicted empirical formula from this study has shown a relationship that could be used in the prediction and estimation of compressive strength of industrial waste materials replacing fine aggregate in concrete, using rebound number. 

Notwithstanding, it is seen that Aliabdo and Elmoaty [26] studied different rocks (marble, white limestone, basalt, pink limestone) and building materials (lime-sand bricks and burned bricks). These materials were cut to testing sizes for compressive strength and rebound hammer tests due to their properties and different behavior in nature. It was seen that they produced different compressive strengths and different rebound numbers and generated different empirical formulas. However, the empirical formula proposed by Aliabdo and Elmoaty [26] was then employed in this study for comparison with the result from theoretical compressive strength calculated using empirical formulas from this study. For the empirical formulas proposed in this study, it can be seen from Table 5 that both theoretical compressive strength (C_30_) and experimental compressive strength (C_30_) have similar compressive strength values with 0.48 as the difference in compressive strength when compared. For AASR5, the difference between theoretical compressive strength and experimental compressive strength is 0.49. This indicates that the empirical formula derived from this study could be used for the prediction of compressive strength.

**Figure 8:**
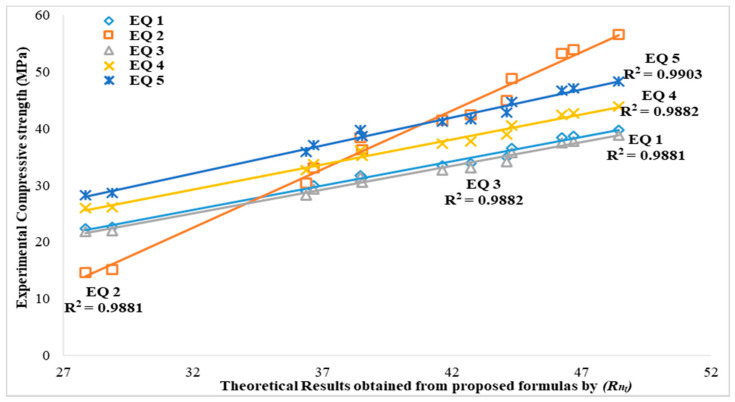
Experimental compressive strength against theoretical compressive strength from the empirical formulas.

A graph of experimental compressive strength against the predicted theoretical compressive strength determined from the empirical formula from this study and the formula adopted from the past study is presented in Figure 8. It is seen from the graph that Equations (1) and (3), an empirical formula proposed by Aliabdo and Elmoaty [26], showed a lower predicted compressive strength value, and it is underestimated among other empirical formulas adopted in this study. Meanwhile, Equation (2) was seen to be between overestimated and underestimated, when compared with the experimental compressive strength and the other theoretical compressive strength from the empirical formula.

Furthermore, the theoretical compressive strength from Equation (5) (predicted formula from this study) showed a strong relationship with the experimental compressive strength. Thus, it can be used for the prediction of compressive strength of industrial waste material (alum sludge, quarry dust and limestone dust) replacing fine aggregate for concrete production.

## 4. Conclusions

The effect of industrial waste material on Schmidt rebound number and the relationship between compressive strength and rebound number is studied in this research. The following conclusions are derived based on the result generated from the experimental work and analysis:

1. All industrial waste materials employed in this study have proved to be good materials for the replacement of fine aggregate in concrete production up to long-term curing age (180 days). Among all the replacement materials, concrete produced with alum sludge waste from a drinking water treatment plant demonstrated impressive results in both destructive and nondestructive test methods.
2. The Schmidt rebound number increases with an increase in industrial waste material and as percentage replacement content of concrete increases. The study also showed that the rebound number value progressively increases with an increase in curing age.
3. The use of Schmidt rebound number for prediction and estimation of compressive strength yields values that are similar to the conventional concrete compressive strength values in this study. Hence, the Schmidt rebound number of the nondestructive test method is a reliable test method for the prediction of compressive strength of concrete.
4. The proposed correlation showed an excellent relationship between rebound number and compressive strength of concrete produced in this study at all curing ages, with correlation coefficients of R^2^ = 0.98, R^2^ = 0.99 and R^2^ = 0.98. Thus, it can be used for the prediction of the compressive strength of concrete.

### Limitations and Future Recommendations

Industrial waste materials employed in this study have shown some level of improvement in concrete strength and improvement in Schmidt rebound number when compared with the control concrete sample in this study. The experiment did not cover the behavior of concrete when alum sludge, quarry dust and limestone dust replace fine aggregate below 5% or above 15%. Therefore, further investigation on nondestructive test by Schmidt rebound hammer test when alum sludge is used at lower fine aggregate replacement content or replacement content above 15% should be done. Further experimental investigation should be conducted on Schmidt rebound hammer test utilizing alum sludge in different treatment temperatures (300, 400, 500 and 600 °C and above), to replace cement in concrete production and to further understand the behavior and effect of the waste on concrete rebound number and predicted strength. Other common nondestructive test methods such as ultrasonic pulse velocity (UPV) should be applied using similar materials. Furthermore, a comparison of the Schmidt rebound hammer test method with other nondestructive methods could be studied in the future using industrial waste materials such as alum sludge, quarry dust and limestone dust.

## Figures and Tables

**Table 1:** Physical properties of materials.

Test	CA	FA	AA	TA	LS	QD
Specific gravity	2.75	2.62	2.38	2.35	2.57	2.58
Water absorption (%)	0.54	0.76	12.52	12.5	1	1.10

CA, coarse aggregate; FA, fine aggregate; AA, air-dried alum sludge; TA, treated alum sludge 200 °C; LS, limestone dust; QD, quarry dust.

**Table 2:** Chemical composition of fine aggregate materials.

Samples	SiO_2_%	Al_2_O_3_%	CaO%	Fe_2_O_3_%	Na_2_O%	K_2_O%	MgO%	MnO%	TiO_2_%
FA	80.96	11.62	3.58	1.78	1.43	1.21	0.77	0.05	-
AA	41.98	33.09	0.43	5.05	0.06	1.83	0.31	0.03	0.58
TA	42.74	33.28	0.43	4.91	-	1.88	0.32	0.02	0.6
LS	13.7	4.8	77.8	3.5	0.9	0.13	1.2	-	-
QD	66.8	17.82	0.84	12.62	-	2.12	4.27	-	-

FA, fine aggregate; AA, air-dried alum sludge; TA, treated alum sludge 200 °C; LS, limestone dust; QD, quarry dust.

**Table 3:** Mix proportion of concrete with different industrial waste materials (kg/m^3^).

S/N	OPC	Coarse Agg.	Fine Agg	Air-Dried Alum Sludge	Treated Alum Sludge	Lime Stone	Quarry Dust	W/C
C_30_	410	1022	679	0	0	0	0	0.52
AASR5	410	1022	648	34	0	0	0	0.52
AASR10	410	1022	614	68	0	0	0	0.52
AASR15	410	1022	580	102	0	0	0	0.52
TASR5	410	1022	648	0	34	0	0	0.52
TASR10	410	1022	614	0	68	0	0	0.52
TASR15	410	1022	580	0	102	0	0	0.52
LSDR5	410	1022	648	0	0	34	0	0.52
LSDR10	410	1022	614	0	0	68	0	0.52
LSDR15	410	1022	580	0	0	102	0	0.52
QDR5	410	1022	648	0	0	0	34	0.52
QDR10	410	1022	614	0	0	0	68	0.52
QDR15	410	1022	580	0	0	0	102	0.52

**Table 4:** Interpretation of rebound hammer test results.

Average Rebound Number	Quality of Concrete
More than 40	Very Good Hard Layer
30 to 40	Good layer
20 to 30	Fair
Less than 20	Poor Concrete
0	Delamination

**Table 5:** Comparison of theoretical and experimental compressive strength using rebound number.

Experimental (MPa)			Theoretical Results Obtained from Proposed Formulas by (*R_nt_*)					Theoretical/Experimental (*R_nt_/f_c_*)				
*S/N*	*R*_n_	*f_c_*	Equation (1)	Equation (2)	Equation (3)	Equation (4)	Equation (5)	Equation (1)	Equation (2)	Equation (3)	Equation (4)	Equation (5)
C_30_	42.45	36.36	28.94	30.43	28.22	32.69	35.88	0.79	0.83	0.78	0.9	0.98
AASR5	43.8	36.64	30.02	33.04	29.28	33.80	37.13	0.82	0.90	0.80	0.92	1.01
AASR10	46.6	38.46	31.78	38.46	31.47	36.11	39.71	0.83	1.0	0.82	0.93	1.03
AASR15	34.25	27.84	22.38	14.58	21.79	25.93	28.30	0.80	0.52	0.78	0.93	1.01
TASR5	45.45	38.53	31.34	36.23	30.57	35.17	38.65	0.81	0.94	0.79	0.91	1.00
TASR10	48.2	41.61	33.54	41.55	32.72	37.43	41.19	0.81	0.99	0.78	0.90	0.99
TASR15	34.55	28.88	22.62	15.16	22.03	26.17	28.60	0.78	0.52	0.76	0.91	0.99
QDR5	48.65	42.7	33.90	42.42	33.08	37.81	41.60	0.79	0.99	0.77	0.88	0.97
QDR10	50	44.11	34.98	45.03	34.14	38.92	42.85	0.79	1.02	0.77	0.88	0.97
QDR15	54.6	46.67	38.66	53.92	37.75	42.71	47.09	0.83	1.15	0.81	0.91	1.00
LSDR5	52	44.31	36.58	48.90	35.71	40.57	44.69	0.82	1.10	0.80	0.91	1.00
LSDR10	54.25	46.24	38.38	53.24	37.47	42.43	46.77	0.83	1.15	0.81	0.92	1.01
LSDR15	56	48.43	39.78	56.63	38.84	43.87	48.39	0.82	1.17	0.80	0.91	0.99

## Data Availability

Not applicable.
